# Lessons from everyday stroke care for clinical research and vice versa: comparison of a comprehensive and a research population of young stroke patients

**DOI:** 10.1186/1471-2377-14-45

**Published:** 2014-03-07

**Authors:** Christian Tanislav, Ulrike Grittner, Bjoern Misselwitz, Gerhard Jan Jungehuelsing, Christian Enzinger, Bettina von Sarnowski, Jukka Putaala, Manfred Kaps, Peter Kropp, Arndt Rolfs, Turgut Tatlisumak, Franz Fazekas, Edwin Kolodny, Bo Norrving

**Affiliations:** 1 Department of Neurology, Justus Liebig University, Klinikstrasse 33, 35392 Giessen, Germany; 2 Center for Stroke Research, Charité – University Medical Centre Berlin, Berlin, Germany; 3 Department for Biostatistics and Clinical Epidemiology, Charité – University Medical Centre Berlin, Berlin, Germany; 4 Institute of Quality Assurance Hesse (Geschäftsstelle Qualitätssicherung Hessen; GQH), Eschborn/Frankfurt, Germany; 5 Department of Neurology, Medical University of Graz, Graz, Austria; 6 Department of Neurology, University Medicine, Ernst Moritz Arndt University, Greifswald, Germany; 7 Department of Neurology, Helsinki University Central Hospital, Helsinki, Finland; 8 Institute of Medical Psychology and Medical Sociology Medical Faculty, University of Rostock, Rostock, Germany; 9 Albrecht-Kossel Institute for Neuroregeneration, University of Rostock, Rostock, Germany; 10 New York University School of Medicine, New York, USA; 11 Department of Neurology, Lund University Hospital, Lund, Sweden

**Keywords:** Ischemic stroke, Stroke in the young, Stroke severity, Stroke registry

## Abstract

**Background:**

Translating knowledge derived from medical research into the clinical setting is dependent on the representativeness of included patients. Therefore we compared baseline data of patients included in a recent large study addressing young stroke in comparison to a large representative stroke registry.

**Methods:**

We analysed baseline data of 5023 patients (age 18-55 years) with an acute cerebrovascular event included in the sifap1 (Stroke in Young Fabry Patients) study. For comparison 17007 stroke patients (age 18-55 years) documented (2004-2010) in a statutory stroke registry of the Institute of Quality Assurance Hesse of the Federal State of Hesse (GQH), Germany.

**Results:**

Among 17007 juvenile (18-55 years) patients identified in the GQH registry 15997 had an ischaemic stroke or TIA (91%) or an intracranial haemorrhage (9%). In sifap1 5023 subjects were included. Sex distribution was comparable (men: 59% sifap1 versus 60.5% GQH) whereas age differed between the groups: median age was 46 years in sifap1 versus 49 years in GQH. Slightly higher percentages for diabetes mellitus and hypertension in the GQH registry were noted. There were no differences in stroke severity as assessed by NIHSS (median 3) and mRS (median 2). In patients with ischaemic stroke or TIA (n = 4467 sifap1; n = 14522 GQH) higher rates of strokes due to small artery occlusion and atherosclerosis occurred in older age groups; cardioembolism and strokes of other determined cause occurred more frequently in younger patients.

**Conclusions:**

The comparison of baseline characteristics between the sifap1 study and the GQH registry revealed differences mainly determined by age.

## Background

Evidence-based medicine is crucial to facilitate appropriate decisions in patient care. It relies on clinical trials, cohort studies, case-control studies, case series, and observational studies. However, different sources of bias might distort results; one of the most important and often discussed confounders is selection bias, which occurs when considering patients for inclusion in a study [1]. Potential reasons for selection bias are strict inclusion criteria determining selected patient-groups, which constitutes only a non-representative proportion of patients of the broad spectrum in the delivery of care [1]. In this context, translating knowledge derived from clinical trials and observational studies into the clinical setting is dependent on the representativeness of the patients included in studies in comparison to the entire population of interest.

Recently, a number of large young stroke studies of single- or multi-centre design has been published. These studies brought valuable data on age and gender distribution, risk factors, etiologic factors, and imaging findings on young stroke patients [2-6]. However, it is crucial to address whether such data are identical with what is seen in daily clinical practice before adapting their conclusions to clinical practice. In this context it is important to consider possible differences between data derived from multinational projects in comparison with nation-wide data collections [2,3].

For this reason, we analysed baseline data of patients included in the recent largest observational young stroke study, namely the sifap1 study and compared with the Hesse Stroke Registry’s data on young stroke patients. Due to the selection process when searching for eligible patients within a general population of reference, differences in baseline characteristics between the two groups could be expected. The nature of the sifap1 study (screening for a rare disease among young strokes) might be a relevant determinant factor.

## Methods

For the analysis two comprehensive prospective stroke populations including young patients (18-55 years) were analysed: (1) sifap1 study and (2) Register data of the Institute of Quality Assurance Hesse (Geschäftsstelle für Qualitätssicherung, GQH) [7,8]. For comparing baseline characteristics the following parameters were considered: sex, age, previous cerebrovascular event, vascular risk factors such as hypertension, diabetes mellitus and current smoking, as well as stroke subtypes according to the Trial of Org 10172 in Acute Stroke Treatment criteria (TOAST) and the stroke severity as assessed by National Institutes of Health Stroke Scale (NIHSS) and modified Rankin Scale (mRS) [9]. In the sifap1 study the NIHSS was determined within 48 h after admission; in GQH the NIHSS was assessed on admission while the mRS was determined one day after admission. 65.7% of the patients included in the sifap1 study are German in origin and 74% derive from German-speaking countries. The protocol for the analysis of the study was reviewed and approved by the ethical committee of the University of Giessen.

### sifap1

The sifap1 study was a prospective multicentre European observational study that aimed to establish the prevalence of Fabry disease in 5023 young patients with a cerebrovascular event (CVE). Patients were recruited between April 2007 and January 2010 at 47 centres in 15 European countries. Inclusion criteria were CVE <3 months prior to enrolment, age 18 to 55 years, and cerebral MRI ≤1 month of inclusion (Table 1). TIA was defined as a CVE with clinical symptoms lasting <24 hours.

**Table 1:** Synopsis with methodological characteristics of the sifap1 study

**Study design**	**Observational prospective cross-sectional multicentre multinational (European) study**
**Study objectives**	
Primary objectives	To determine the prevalence of Fabry disease in young stroke patients
Secondary objectives	To describe patterns of stroke in young patients
**Inclusion criteria**	
Age	18–55 years
Time since event	Less than 3 months before inclusion into the study
Diagnosis	Acute CVE of any aetiology (ischemic stroke, TIA, intracranial haemorrhage)
Verification of diagnosis	Verification of brain infarction or haemorrhage by MRI scan. In case of negative MRI diagnosis confirmed by stroke-experienced neurologist (more than 2 years of experience in stroke and at least 6 years of experience in general neurology)
Diagnostic information	MRI documentation available Diagnostic procedures according to EUSI/ESO recommendations
Ethics	Written informed consent from patient or legal representative according to local ethics committee regulations
**Inclusion period**	April 2007 till January 2010
**Number of participating countries**	15
**Number of centres**	47
**Number of included patients**	5023
**Overall inclusion rate in the 7 best recruiting centres**	52.9%

Besides genetic issues the study protocol directed a detailed documentation of parameters such as vascular risk factors, stroke severity, laboratory values, and results of the diagnostic work up. All patients or their legal representatives provided written informed consent. The study was performed according to the Helsinki Declaration and has been approved by all local ethical committees of the participating centres [2,7].

### GQH

The GQH database is an obligatory federal-state-wide hospital-based registry that covers more than 95% of all ischaemic strokes, transient ischaemic attacks (TIA) and intracerebral haemorrhages in a community of more than 6 million inhabitants of the Federal State of Hesse (Germany). TIA was defined as a CVE with clinical symptoms lasting <24 hours. The GQH-data include various parameters of acute in-patient care, as well as factors proved to be relevant for the course and the prognosis of stroke. The acquisition of data for quality assurance reasons is regulated by law and implemented as a guideline, which is elaborated by the Federal Joint Committee for hospital quality assurance in accordance with Volume V of the Social Insurance Code (§137 SGB V and §135a SGB V). Based on this regulation, the Hesse State Hospital Law contains a provision that allows the GQH to record such data legally. The publication of aggregate quality assurance data has also been cleared with the Hesse Data Protection Commissioner, so no data protection problem arises here either [10,11].

### Statistical analysis

Continuous variables were analysed by calculating the median and mean value and the interquartile range (25%-percentile and 75%-percentile). Nonparametric data were analysed employing the Mann–Whitney U-test. For comparing relative frequencies Chi-squared test was used. All statistical analyses were performed with the PASW Statistics 18, release version 18.0.2 (© SPSS, Inc., 2009, Chicago, IL, http://www.spss.com) and SAS software, version 9.2 of the SAS System for Windows (© 2008 SAS Institute Inc., Cary, NC).

## Results

**Figure 1:**
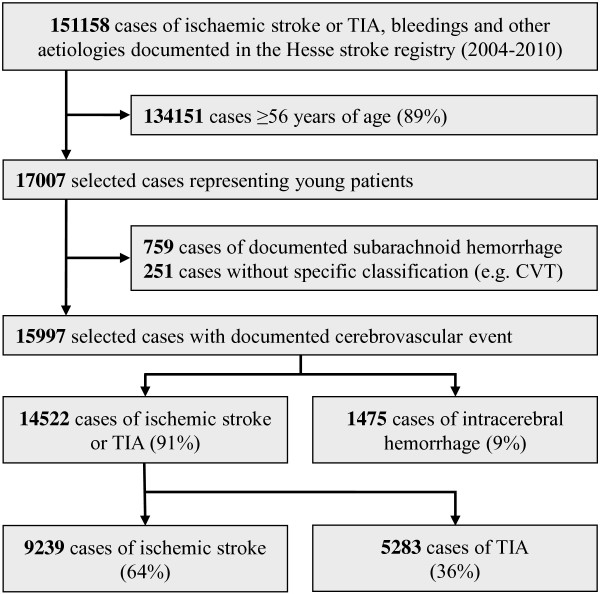
Patients selection within the stroke registry of the Institute of quality assurance Hesse (GQH)

Among 151,158 documented cases between 2004 and 2010 in the Hesse Stroke Registry 17,007 cases were identified as juvenile (age < 56 years) and 15,997 had a cerebrovascular event (91% ischaemic stroke or TIA, 9% intracerebral haemorrhage) (Figure 1). In the sifap1 study 5023 subjects were included; 23.3% had TIA, 70.7% brain infarction, and 5.6% haemorrhagic strokes.

**Figure 2:**
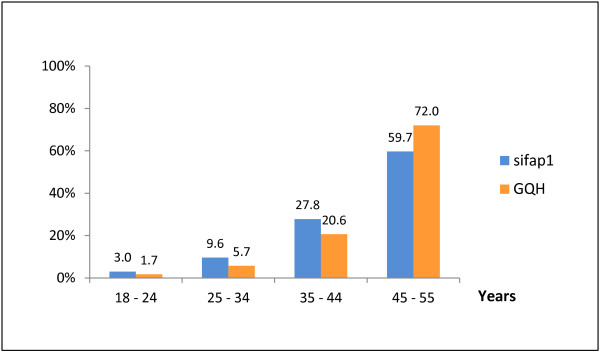
Age distribution stratified by age classes in sifap1 (n = 5023) versus GQH (n = 17007)

The sex distribution in both groups was comparable (males 59% sifap1 versus 60.5% GQH). There was an obvious difference in the age distribution between the two groups (Figure 2). The proportion of younger patients in sifap1 was higher, whereas in the age category 45 to 55 years a higher percentage of patients within GQH was noted compared to sifap1 (72% versus 59.7%; Figure 2).

Brain infarctions totalled in sifap1 a higher proportion of patients than in the GQH group (70.7% versus 54.3%, p < 0.001). While the distribution of vascular risk factors in the two groups revealed slightly higher percentages for diabetes mellitus and hypertension in the GQH group, the stroke severity as determined by NIHSS and mRS were identical regarding median and interquartile ranges (Table 2).

**Table 2:** Baseline characteristics, risk factors, and stroke severity

	**sifap1** **(n = 5023)**	**GQH** **(n = 17007)**
Sex (males) (n = 5023, sifap1)	59%	60.5%
Age (years); median and 25th-75th percentile	46 (40-51)	49 (44-53)
Previous (symptomatic) cerebrovascular event (n = 5022, sifap1)	16.8%	10.9%***
Event entities		
TIA	22.3%	31.1%***^a^
Brain infarction	70.7%	54.3%
Primary haemorrhage	5.6%	8.7%
Others†	1.4%	5.9%
Missing cases	217	-
Vascular risk profile		
Hypertension (n = 4994, sifap1)	47%	49%*
Diabetes (n = 4996, sifap1)	10%	13.3%***
Current smoking (n = 5022, sifap1)	41.4%	38.5%***
Stroke severity		
NIH stroke scale (median; 25th-75th percentile)	3 (1-6)^║^	3 (1-6)‡
Modified rankin scale (median; 25th-75th percentile)	2 (1-3)^║^	2 (1-3)§

†consider subarachnoid haemorrhage and cerebral sinus or vein thrombosis.

^║^assessed within the first 48 hours.

‡assessed on admission.

§assessed one day after admission.

*p < 0.05.

**p < 0.01.

***p < 0.001.

^a^the test statistic was calculated by using all entity categories.

In the subgroups of patients with ischaemic stroke or TIA the sex distribution remained similar as compared in the total groups (59.4% males in sifap1 versus 61.2% GQH, Table 3). Considering patients with ischaemic stroke or TIA, the difference in age distribution persisted between the two groups with a three years shift (median 47 years sifap1 versus 50 years GQH). The proportion of TIA patients was higher in GQH (36.4%) than in sifap1 (24%); more patients with a previous stroke were included in sifap1 (16.9% versus 11.9%). In the age category of 45-55 years the proportion of patients with hypertension was similar (58.2%) in both groups (Table 3).

**Table 3:** Subgroup of ischaemic strokes or TIA stratified by age groups (demographic characteristics, risk factors, and stroke subtypes)

	**Total groups**		**Age 18-34 years**		**Age 35-44 years**		**Age 45-55 years**	
	**sifap1(n = 4467)**	**GQH(n = 14522)**	**sifap1(n = 563)12.6%**	GQH(n = 1049)7.2%***	**sifap1(n = 1224)27.4%**	GQH(n = 2962)20.4%***	**sifap1(n = 2680)60%**	GQH(n = 10511)72.4%***
Sex (males); (n = 4467, Sifap1)	59.4%	61.2%*	42.8%	46.3%	57.9%	57.9%	63.5%	63.5%
Age (years); mean, median and 25th-75th percentile (n = 4467, Sifap1)	44.747 (40-51)	47.5*** 50 (44-53)	28.329 (25-32)	28.4 29 (25-32)	40.541 (38-43)	40.6 41 (39-43)	50.150 (47-53)	51.4*** 51 (48-53)
Previous cerebrovascular event (n = 4466, Sifap1)	16.9%	11.9%***	12.8%	7.7%**	15.4%	8.2%***	18.6%	13.3%***
Proportion of TIA (n = 4467, Sifap1)	24.0%	36.4%***	21.8%	41.5%***	25.1%	39.7%***	23.9%	34.9%***
Vascular risk profile								
Hypertension (n = 4439, Sifap1)	46.6%	49.5%**	13.1%	11.6%	36.7%	31.7%**	58.2%	58.2%
Diabetes mellitus (n = 4443, Sifap1)	10.3%	14.4%***	2.0%	3.1%	7.7%	8.6%	13.3%	17.1%***
Current smoking (n = 4466, Sifap1)	42.5%	40.8%*	37.5%	33.5%	42.1%	41.3%	43.8%	41.5%
Stroke subtypes according to TOAST	**n = 4345**	**n = 14169**	**n = 555**	**n = 1006**	**n = 1201**	**n = 2902**	**n = 2589**	**n = 10261**
Atherosclerosis	16.4%	22.3%***^a^	4.1%	8.8%***^a^	10.4%	13.6%***^a^	21.8%	26%***^a^
Cardiac embolic source	15.1%	12.1%	18.6%	15.3%	17.7%	13.4%	13.2%	11.4%
Small artery occlusion	13.7%	19.2%	6.3%	8.2%	9.5%	13.7%	17.2%	21.8%
Other determined cause	16.5%	9.8%	24.5%	16.7%	20.8%	14.3%	12.8%	7.8%
Undetermined cause	38.3%	36.7%	46.5%	51.0%	41.6%	45.0%	34.9%	33.0%

*p < 0.05.

**p < 0.01.

***p < 0.001.

^a^the test statistic was calculated by using all five TOAST classification categories.

**Figure 3:**
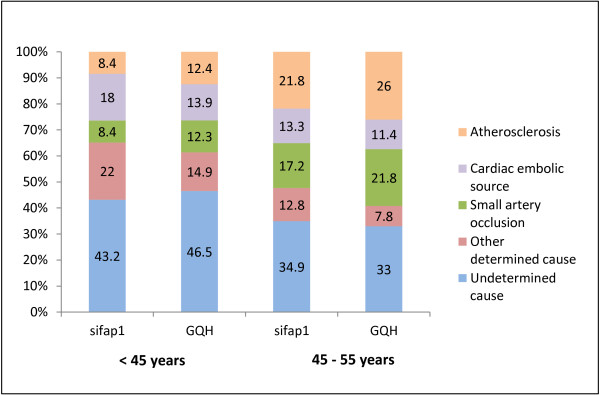
Stroke subtypes stratified by age sifap1 versus GQH

The distribution of stroke subtypes revealed a high percentage of patients classified as having an ischaemic stroke of other determined or undetermined cause in the sifap1 study (Table 3). In older age groups (comparing 45-55 years versus 18-44 years) higher rates of strokes due to small artery occlusion (GQH: 21.8% versus 12.3% and Sifap 1: 17.2% versus 8.4%) and atherosclerosis (GQH: 26% versus 12.4% and sifap1: 21.8% versus 8.4%) were obvious; rates of cardiac embolic strokes (GQH: 11.4% versus 13.9% and sifap1: 13.3% versus 18%) and strokes of other determined cause (GQH: 7.8% versus 14.9% and sifap1: 12.8% versus 22%) lowered from the young to the older age category in both populations (Table 3 and Figure 3).

## Discussion

The main difference between the sifap1 and the corresponding GQH group represents the more left skewed age distribution in the GQH group as compared to sifap1: in sifap1 more younger patients were included, while in the GQH a higher proportion of patients aged 45-55 were evident. This result is in line with previous investigations, revealing comparable differences when considering the entire population of young stroke patients in sifap1 centres compared to those patients included in sifap1 [7].

However, in both groups (sifap1 and GQH) the age distribution is skewed to the left: when creating smaller age categories the proportions of patients were higher in older age groups (Figure 2). In accordance with previous investigations in both populations higher percentages of vascular risk factors in elderly patients were observed [2,3,12,13]. In this context the markedly higher proportion of vascular risk factors in the GQH registry might partially be determined by the higher proportion of older patients in the registry as compared to sifap1. In the sifap1 study, which specifically addressed stroke in the young, a selection bias for recruiting particularly younger patients could be suspected. In contrast in the GQH this phenomenon is of minor importance, as the registry regards to the entirety of strokes without any restrictions.

In the subgroups of patients with ischaemic stroke or TIA the difference in age might determine the distribution of aetiologies as well. In older aged patients lower proportions of cardioembolic strokes, strokes of other determined cause or undetermined cause were obvious, whereas proportions of stroke causes, such as atherosclerosis and small artery occlusion were higher in older aged patients. The higher age level in the GQH affected differences in the prevalence of stroke aetiologies between sifap1 and GQH data (Figure 3). Nevertheless the distributions of aetiologies in both collectives seem comparable with a consistent association between age and prevalence differences. This underlines the reliability of the data accumulated in the sifap1 study and GQH registry, respectively.

In the subgroup of patients with ischaemic stroke or TIA the proportion of previous cerebrovascular events in the sifap1 study was markedly higher than in the GQH data (16.9% versus 11.9%). The proportion of TIAs is higher in the GQH-data than in sifap1. Regarding the very young subgroups (18-44 years), in sifap1 the proportion of patients with a previous cerebrovascular event doubled the corresponding value in GQH (14.5% versus 7.7%).

In young stroke patients with a previous cerebrovascular event and predominantly without vascular risk factors and with unknown cause for stroke, considering Fabry disease as a potential differential diagnosis appears mandatory, and might facilitate the inclusion in sifap1 a welcome option [14]. On the other hand previous cerebrovascular events might be missed in a global registry due to less rigorous requirements regarding imaging procedures and data acquisition. For inclusion in the sifap1 study a cerebral MRI scan was mandatory. In the GQH registry in 73% of patients with ischaemic stroke or TIA underwent the procedure during the acute treatment (unpublished data considering patients (n = 4783) documented 2009-2010 in the GQH registry; for these two years a consistent documentation of cerebral MRI was available). This might also determine the higher proportion of patients diagnosed with a TIA in the GQH registry.

Comparing the entire populations, the proportion of brain haemorrhages in the GQH stroke registry ranged higher (8.7% versus 5.6%) than in sifap1. The restraint in including bleedings in sifap1 might be also determined by the nature of the study, as cerebral haemorrhages are rarely accompanied by Fabry disease [15,16].

The evaluation of the disability data rendered similar results in sifap1 and GQH; both scales (mRS, NIHSS) showed identical median and interquartile values. With regard to stroke severity, the populations are therefore comparable. Considering that in clinical trials or observational studies severely disabled patients are less selected for inclusion, due to difficulties in handling, in sifap1 this aspect was obviously less relevant. In conclusion, concerning the stroke severity the sifap1 study is adequately balanced with distributions approaching the situation in the care delivery as reflected in the GQH stroke registry.

Similarly to the stroke severity, the sex distribution is comparable in both groups, with somewhat higher proportions of women in sifap1. In older age the proportion of males is higher than in younger age groups. Among the oldest age range (45-55 years) the vascular disease might preponderate in males more than in females, determining more strokes related to a vascular burden such as atherosclerotic strokes or strokes due to small artery occlusion among males [3,17]. When including patients in a clinical study addressing stroke or a rare disease causing stroke the patient’s sex per se could not be expected to influence on the behaviour regarding inclusion. Therefore the almost identical sex distribution in both groups renders a reciprocal validation in the reliability of the accumulated data in sifap1 and GQH, respectively.

## Conclusion

Differences in baseline characteristics between the sifap1 study and the GQH stroke registry were noted. They were mainly determined by age. In these specific patient groups including young strokes (≤55 years) the median age in the sifap1 study was 3 years below the median in the GQH. Stroke aetiologies determined by vascular burden were more often present in older patients, whereas undetermined stroke causes and stroke of other determined aetiologies occurred less frequent. Especially among the young groups (18-44) high proportions of cryptogenic strokes were evident in the sifap1 study as well as in the GQH stroke registry (43% and 46%). Comparable sex distributions in sifap1 and GQH, in this particular case a factor robust against selection bias, underline the reliability of the data accumulated in both collectives.

Sifap1 represents a pivotal study on young stroke providing important information on distribution of risk and etiologic factors. One of the novel findings depicted in sifap1 is the high prevalence of vascular risk factors in young stroke patients [2]. In this context, the comparison with a comprehensive population is of high relevance and need to be taken into consideration when referring to sifap1. It indicates, that in a general population of young strokes, even higher percentages of vascular risk factors (hypertension and diabetes) and stroke aetiologies related to a vascular burden (arteriosclerosis and small artery occlusion) might be expected. It emphasises the need of new strategies in the primary stroke prevention considering the substantial contribution of vascular risk factors for causing strokes in young age.

## Competing interest

The authors declare that they have no conflicts of interest that may be relevant to the submitted work. All authors have completed the Unified Competing Interest form at http://www.icmje.org/coi_disclosure.pdf (available on request from the corresponding author).

## Authors’ contributions

The present analysis was conducted by UG and CT. All authors were involved in the analysis and interpretation of the data and in drafting the manuscript. All authors read and approved the final manuscript.

## Pre-publication history

The pre-publication history for this paper can be accessed here:

http://www.biomedcentral.com/1471-2377/14/45/prepub
